# Supplementary material for: Resolving the full spectrum of human genome variation using Linked-Reads
Source: Genome Res. 2019 Apr;29(4):635–45. doi: 10.1101/gr.234443.118 (PMC6442396; doi:10.1101/gr.234443.118)
Supplement: Supplemental Material [file supp_gr.234443.118_Supplemental_longranger-2.2.zip › longranger-2.2.tar.bz2!/lr2.2-public/lib/stan/cmdstan/stan/doc/api/html/functions_vars_s.html]

Stan: Class Members - Variables


|  |  |
| --- | --- |
|  | Stan  2.5.0  probability, sampling & optimization |


- Main Page
- Related Pages
- Namespaces
- Classes
- Files

- Class List
- Class Index
- Class Hierarchy
- Class Members

- All
- Functions
- Variables
- Typedefs
- Enumerator
- Related Functions

- \_
- a
- b
- c
- d
- e
- f
- g
- h
- i
- k
- l
- m
- n
- o
- p
- q
- r
- s
- t
- u
- v
- w
- x
- y
- z


All Classes Namespaces Files Functions Variables Typedefs Enumerations Enumerator Friends Macros Pages

### - s -

- s\_bar\_
  : stan::mcmc::stepsize\_adaptation
- sample\_r
  : stan::gm::statement\_grammar< Iterator >
- samples
  : stan::io::stan\_csv
- sampling
  : stan::io::stan\_csv\_timing
- save\_warmup
  : stan::io::stan\_csv\_metadata
- seed
  : stan::io::stan\_csv\_metadata
- sign
  : stan::mcmc::nuts\_util
- simplex\_decl\_r
  : stan::gm::var\_decls\_grammar< Iterator >
- size\_
  : stan::agrad::op\_matrix\_vari
  , stan::agrad::op\_vector\_vari
  , stan::agrad::stored\_gradient\_vari
  , stan::math::coupled\_ode\_system< F, double, double >
  , stan::math::coupled\_ode\_system< F, double, stan::agrad::var >
  , stan::math::coupled\_ode\_system< F, stan::agrad::var, double >
  , stan::math::coupled\_ode\_system< F, stan::agrad::var, stan::agrad::var >
- stage\_
  : stan::gm::var\_size\_validating\_visgen
- stan\_version\_major
  : stan::io::stan\_csv\_metadata
- stan\_version\_minor
  : stan::io::stan\_csv\_metadata
- stan\_version\_patch
  : stan::io::stan\_csv\_metadata
- statement\_
  : stan::gm::for\_statement
  , stan::gm::program
  , stan::gm::statement
- statement\_2\_g
  : stan::gm::statement\_grammar< Iterator >
- statement\_2\_r
  : stan::gm::statement\_2\_grammar< Iterator >
- statement\_g
  : stan::gm::functions\_grammar< Iterator >
  , stan::gm::program\_grammar< Iterator >
  , stan::gm::statement\_2\_grammar< Iterator >
- statement\_r
  : stan::gm::statement\_grammar< Iterator >
- statement\_seq\_r
  : stan::gm::statement\_grammar< Iterator >
- statements\_
  : stan::gm::statements
- step\_size
  : stan::io::stan\_csv\_adaptation
- stepsize\_adaptation\_
  : stan::mcmc::stepsize\_adapter
  , stan::mcmc::stepsize\_covar\_adapter
  , stan::mcmc::stepsize\_var\_adapter
- subject
  : stan::gm::unary\_op
- sum\_prob
  : stan::mcmc::nuts\_util
- system\_function\_name\_
  : stan::gm::integrate\_ode


---

[ Stan Home Page ]

*© 2011–2014,
Stan Development Team.*
